# Dietary Cadmium Intake and Risk of Breast, Endometrial and Ovarian Cancer in Danish Postmenopausal Women: A Prospective Cohort Study

**DOI:** 10.1371/journal.pone.0100815

**Published:** 2014-06-25

**Authors:** Kirsten T. Eriksen, Jytte Halkjær, Mette Sørensen, Jaymie R. Meliker, Jane A. McElroy, Anne Tjønneland, Ole Raaschou-Nielsen

**Affiliations:** 1 Danish Cancer Society Research Center, Copenhagen, Denmark; 2 Department of Preventive Medicine and Graduate Program in Public Health, Stony Brook University, Stony Brook, New York, United States of America; 3 Family and Community Medicine, University of Missouri, Columbia, Montana, United States of America; Indiana University, United States of America

## Abstract

**Purpose:**

Cadmium is a human lung carcinogen and possesses estrogen-like activity. This combination of carcinogenic and estrogenic activity makes cadmium a contaminant of high concern for hormone-related cancers. Diet and smoking are the main sources of cadmium exposure. The aim of this study was to investigate the association between dietary cadmium intake and risk of breast, endometrial and ovarian cancer in Danish postmenopausal woman.

**Methods:**

We estimated dietary cadmium intake in the Diet, Cancer and Health cohort at enrolment 1993-97. The estimates were based on food frequency questionnaires and cadmium contents in all foods. Among 23,815 postmenopausal women we identified 1390 breast, 192 endometrial, and 146 ovarian cancer cases from enrolment through December 31, 2010 using the Danish Cancer Registry. Cox regression was used to analyse the association between dietary cadmium intake and cancer risk.

**Results:**

Mean dietary cadmium intake was 14 µg/day. Cadmium was not associated with breast cancer, incidence rate ratio (IRR) = 0.99, 95% confidence interval (CI): 0.87–1.13 per 10 µg higher dietary cadmium intake/day; endometrial cancer, IRR = 1.08, 95% CI: 0.76–1.53; or ovarian cancer, IRR = 1.15, 95% CI: 0.78–1.70. We found a positive association between cadmium and endometrial cancer for the women with BMI<25 (IRR = 1.50, 95% CI: 0.94–2.39), whereas an inverse association was seen for the women with BMI≥25 (IRR = 0.69, 95% CI: 0.42–1.12); *p* value for interaction  = 0.02.

**Conclusions:**

Our study does not indicate that our estimated dietary cadmium intake is associated with hormone-related cancers in women.

## Introduction

Breast cancer is the most frequently diagnosed cancer among women in Denmark and endometrial and ovarian cancer are the most commonly diagnosed gynaecological cancers. These three cancers are the most frequent hormone-related cancers among women, collectively accounting for about 6000 new cases of cancer in Denmark each year [1].

Established risk factors of these female cancers are related to cumulative exposure to estrogens and reproductive life such as early age at menarche, nulliparity, late age at first pregnancy, short lactation and late menopause [2]. Use of hormone replacement therapy (HRT), higher BMI, lower physical activity, and a family history of the disease are also recognized risk factors for these cancers [2]–[3]. In addition, breast cancer is also related to higher alcohol consumption, radiation exposure, higher educational level, and a higher socioeconomic status [2]–[3]. However, one study has shown that four of the most well-established risk factors for breast cancer (later age at first birth, nulliparity, higher family income, and first-degree family history of breast cancer) still may explain less than half of the breast cancer cases in the United States [4].

Cadmium is an IARC classified group 1 proven human carcinogen of the lung based on mechanistic and epidemiologic evidence from high-exposure occupational settings [5]. Proposed mechanisms of cadmium carcinogenesis include oxidative stress, DNA damage, altered DNA repair, and enhanced proliferation and/or depressed apoptosis [6]–[7]. More recently, both *in vitro* and *in vivo* studies have demonstrated that cadmium also exerts estrogenic activities, such as proliferation of breast cancer cells [8]–[10], activation and increased expression of estrogen regulated genes [9], [11] and activation of the estrogen receptor (ER)-α [9]–[10], [12]–[13]. Animal experiments have shown that environmentally relevant doses of cadmium induce estrogenic responses in female rats, including increased uterine weight and hypertrophy of the endometrial lining with these responses blocked by anti-estrogen [14]. This combination of carcinogenic and estrogenic activities makes cadmium a contaminant of particularly high concern for hormone-dependent cancers. Further, cadmium has been shown to induce progesterone receptor (PGR) levels in breast cancer cells, the induction being blocked by anti-estrogen [9]. Mechanistic and epidemiologic evidence suggest that estrogen-mimicking contaminants, including the environmental and dietary pollutant cadmium, may contribute to development of hormone-related cancers [14].

Diet is a main source of human exposure to cadmium in the non-occupationally exposed population. Cereal products and vegetables are important dietary exposure sources [15]–[16], as plants absorbs cadmium from phosphate fertilizer and fallout due to fossil fuel and waste combustion and due to the high consumption of these dietary items. Smoking is also an important source of cadmium exposure, since cadmium easily accumulates in the tobacco plant and cadmium in tobacco smoke is effectively absorbed in the lungs. The average cadmium intake from food generally varies from 8–25 µg/day [17] and daily cadmium exposures can double in smokers [18].

Three prospective cohort studies have assessed the association between dietary cadmium intake and risk of breast cancer, of which one study indicated an association [19], whereas two did not report any associations [20]–[21]. The authors of the former study also investigated dietary cadmium exposure in relation to endometrial cancer, showing a significant positive association [22], whereas they did not find an association for ovarian cancer [23].

The present study aimed to investigate whether dietary cadmium exposure is associated with hormone-related cancer of the breast, endometrium and ovary assessed in a large population-based prospective cohort in Denmark.

## Materials and Methods

### Ethics Statement

This study was approved by the regional research ethic committee for Copenhagen and Frederiksberg. Written informed consent was obtained from all study participants. The study was carried out without contact to the cohort members or their families. Anonymity of participants was retained by strict data management.

### Study Population

From December 1, 1993, through May 31, 1997, a total of 57,053 individuals (29,875 women and 27,178 men), who were aged 50–65 years, born in Denmark, and had no previous cancer diagnosis, were enrolled in the prospective Diet, Cancer and Health cohort [24]. At enrolment, each participant completed a self-administered, interviewer-checked 192 item semi-quantitative food frequency questionnaire and a questionnaire covering lifestyle habits including information on present and previous smoking, physical activity, reproductive history, health status and social factors. In total 56,999 persons filled in the detailed dietary questionnaires.

We used the Danish Cancer Registry to identify incident cases of invasive breast cancer, endometrial cancer and ovarian cancer among cohort members. Information on ER status, PGR status and histology type was obtained from The Danish Breast Cancer Cooperative Group (DBCG) [25]. To limit the impact of endogenous estrogens and thereby to avoid masking the potential estrogenic influence of cadmium, we restricted our study to women postmenopausal at baseline. Data on potential confounders were obtained from the detailed questionnaires administered at enrolment. 23,815 postmenopausal women (1390 breast cancer cases, 192 endometrial cancer cases and 146 ovarian cancer cases) had complete covariate information and were used for statistical analyses. Mean follow-up time was 13 years for the cohort.

### Assessment of Dietary Cadmium Exposure

We estimated dietary cadmium intake per day for each person in the prospective DCH cohort based on the 192 item semi-quantitative food frequency questionnaire filled in at enrolment. For the calculations we used food monitoring data from The Danish Food Monitoring Programme for Nutrients and Contaminants, 1993–97 [26]. The Danish Food Monitoring Programme was initiated in 1983 and monitoring cycles run for 5-year periods to allow for a comparison of trace element contents (including cadmium) over time in food items sold in Denmark and to assess the potential health concerns of the dietary intake of the trace elements investigated. The samples of each food item were analysed individually, giving detailed information on the variation of trace elements in food items sold on the Danish market. The number of samples analysed of each specific food item was decided on the basis of earlier experience concerning the variation in contents of trace elements in that specific food item. For our study, dietary cadmium measurements from the 5-year monitoring period 1993–97 were used, since this period matches with the period of completion of the food frequency questionnaire in the DCH cohort. The contents of more than 80 different foods were monitored from 1993–97. For food items where data were not available during this period, we used data from the monitoring period 1998–2003, and data from unspecified years. The obtained cadmium concentration for each food item was added to the food table using the FoodCalc program [27] and we obtained an estimate of dietary cadmium intake per day (µg cadmium per day) for each participant in the DCH cohort.

### Statistical Analyses

We used Cox proportional hazard models with age as the underlying time scale [28]. This ensured comparison of individuals of the same age. We used left truncation at age of enrolment, so that people were considered at risk from enrolment into the cohort, and right censoring at the age of cancer under study (event), death, emigration, any other cancer diagnosis (except non-melanoma skin cancer), or end of follow-up (December 31, 2010), whichever came first.

We estimated crude and adjusted incidence rate ratios (IRRs) using the estimate of dietary cadmium intake as a linear variable. The adjusted models were carried out with *a priori* defined potential confounders: Educational level (<8 y; 8–10 y; >10 y) as a measure of socioeconomic status, smoking status (never; former; current), as smoking is a major source of cadmium and never smokers are at lower risk of endometrial and ovarian cancer, and the following known risk factors: number of births (0; 1–2; 3–8), age at first birth (years, continuous), HRT status (never; former; current), HRT use (years, continuous), age at menarche (years, continuous), BMI (continuous), height (cm, continuous), physical activity (MET score, continuous), and alcohol intake (g/day, continuous). Linearity was evaluated with use of linear splines with three boundaries for dietary cadmium intake, age at first birth, HRT use, age at menarche, BMI, height, physical activity and alcohol intake and there was no deviation from linearity. Also, we estimated crude and adjusted IRRs for tertiles of daily dietary cadmium intake, based on distribution among all cohort members. Only participants with complete covariate information were included. Only adjusted IRRs are reported here, as crude and adjusted IRRs were similar in all analyses.

Further, we evaluated *a priori* specified individual characteristics as potential effect modifiers (for breast and endometrial cancer): Educational level (<8, 8–10 y, >10 y), smoking status (never, former, present), HRT use (never, former, present), BMI (<25, ≥25), dietary zinc intake (<median, ≥median), and dietary iron intake (< median, ≥median).

In order to minimize the potential effect of exposure to endogenously produced adipose tissue estrogen, obtained hormones from medical treatment and/or to smoking-derived cadmium, we also restricted analyses to participants who: 1) were never smokers and had BMI<25, and 2) were never smokers and never HRT users, 3) had BMI<25 and were never HRT users, and 4) were never smokers, had BMI<25 and were never HRT users. This was only analysed for breast cancer due to the relatively small number of endometrial and ovarian cancer cases.

Also, we calculated IRRs for different subgroups of breast cancer classifications: Estrogen receptor status (ER+ and ER-), progesterone receptor status (PGR+ and PGR-) and the two most frequent histology types (ductal and lobular).

The procedure PHREG in SAS version 9.3 (SAS Institute, Cary, North Carolina, USA) was used for the statistical analyses.

## Results

Among the 29,875 women of the Diet, Cancer and Health cohort, we excluded 338 with a cancer diagnosis before baseline, 1 with unknown month of cancer diagnosis, 25 with no dietary cadmium exposure data, 5,295 not being defined as postmenopausal at baseline, and 401 with incomplete covariate data. This resulted in a study population of 23,815 postmenopausal women with complete covariate data.

The mean estimated daily cadmium intake of the cohort was 14 µg/day (5–95% percentiles  = 8–22 µg cadmium/day). No major difference in dietary cadmium intake was observed between cancer cases and cohort. As expected, breast and endometrial cancer cases seemed to be higher educated than the cohort and ovarian and endometrial cancer cases were more likely to be never smokers compared with the cohort. In general, cases were more likely to be current users of hormone replacement therapy and to be nulliparous, compared with the cohort (Data not shown).

In our study population, cereal products and vegetables, including potatoes, together contributed to the majority of the estimated dietary cadmium exposure with a mean (SD) of 82% (8%). Specifically, whole grain products contributed a mean (SD) of 33% (12%), potatoes 20% (9%), vegetables, exclusive potatoes, 17% (8%) and refined cereal products 12% (7%). In contrast, meat (red meat, poultry and processed meat), fish, fruit and dairy products contributed only with a mean (SD) of 2.2% (1.5%), 1.8% (1.2%), 2.3% (0.2%), and 0.2% (0.2%), respectively, of the mean cadmium intake (data not shown). Table 1 illustrates relevant characteristics at baseline by tertiles of dietary cadmium intake, based on the cohort. The proportion of women with high educational level increased with increasing dietary cadmium exposure whereas the opposite patterns were seen for women with low educational level (Table 1). Also, the proportion of never smoking women increased with increased dietary cadmium exposure whereas the opposite was seen for current smoking women. Higher whole grain intake and vegetable intake were associated with higher dietary cadmium intake. Also height, physical activity, energy intake, zinc intake, and iron intake increased with increasing tertiles of dietary cadmium intake, whereas alcohol intake slightly decreased.

**Table 1:** Baseline characteristics by tertiles of dietary cadmium intake in the cohort (N = 23,815) of the Diet, Cancer and Health Study, 1993–97.

	Tertiles of dietary cadmium intake		
	<11.9 µg/day	11.9–15.3 µg/day	>15.3 µg/day
Age (years)	57 (57)	57 (57)	57 (57)
Education (years), %			
Low (<8)	39	33	29
Medium (8–10)	49	50	48
High (>10)	12	17	23
Smoking, %			
Never	37	43	47
Former	21	24	26
Current	42	33	27
Hormone replacement therapy (HRT) status, %			
Never	50	50	48
Former	34	34	36
Current	16	16	16
Number of births, %			
0	12	12	13
1–2	60	60	59
3–8	28	28	28
Age at first birth^a^	23 (23)	24 (23)	24 (24)
Years of HRT use^b^	6 (4)	6 (4)	6 (4)
Age at menarche	14 (14)	14 (14)	14 (14)
BMI (kg/m^2^)	26 (25)	26 (25)	25 (25)
Height (cm)	163 (163)	164 (164)	165 (165)
Physical activity (MET h/week)	62 (53)	68 (59)	75 (66)
Energy intake (kcal/day)	1,650 (1,636)	2,047 (2,022)	2,533 (2,468)
Alcohol (g/day)^c^	15 (10)	14 (10)	13 (9)
Zinc intake (mg/day)^d^	14 (12)	17 (14)	20 (18)
Iron intake (mg(day)^d^	14 (10)	16 (13)	19 (16)
Whole grain intake (g/day)	27 (24)	38 (34)	51 (48)
Vegetable intake (g/day)	128 (118)	179 (168)	256 (237)

a Among those having given birth.

b Among evers users (those reporting use for at least one year).

c Among drinkers.

d Sum of intake from diet and supplement.

Mean (median) values are given if not otherwise specified.

In general, we did not observe significant associations between dietary cadmium intake and cancer risk for any of the three investigated hormone-related cancers (Table 2), neither in linear nor categorical analyses. For ovarian cancer there seemed to be a tendency towards a positive association, but confidence intervals were wide due to the relatively low number of cases, and estimates were not statistically significant.

**Table 2:** Incidence rate ratios of breast, endometrial, and ovarian cancer and for the two upper tertiles (T2 and T3) of cadmium exposure compared with lowest tertile (T1, Referent).

Cancer site	Tertile	N cases	IRR^a^	95% CI
Breast	per 10 µg Cd	1390	0.99	(0.87, 1.13)
	T1	468	Referent	
	T2	461	0.96	(0.85, 1.10)
	T3	461	0.97	(0.85, 1.11)
Endometrium	per 10 µg Cd	192	1.08	(0.76, 1.53)
	T1	60	Referent	
	T2	67	0.97	(0.69, 1.37)
	T3	65	0.83	(0.58, 1.19)
Ovary	per 10 µg Cd	146	1.15	(0.78, 1.70)
	T1	45	Referent	
	T2	50	1.14	(0.76, 1.71)
	T3	51	1.20	(0.79, 1.81)

Abbreviations: IRR, incidence rate ratio; CI, confidence interval; Cd, cadmium.

In the linear analyses, dietary cadmium was entered as a continuous variable; IRRs were estimated per 10 µg/day higher intake in dietary cadmium.

Age is underlying time-scale.

a Adjusted for educational level (<8 y; 8–10 y; >10 y), smoking status (never; former; current), number of births (0; 1–2; 3–8), age at first birth (years, continuous), HRT status (never; former; current), HRT use (years, continuous), age at menarche (years, continuous), BMI (continuous), height (cm, continuous), physical activity (MET score, continuous) and alcohol intake (yes/no and g/day, continuous).

Adjusted model results are shown.

Table 3 shows risk estimates for potential effect modifiers for breast cancer and endometrial cancer. We did not complete these analyses for ovarian cancer due to the relatively low number of cases that would be included in each stratum. BMI seemed to modify the association between cadmium intake and endometrial cancer as we found a positive association for the group with BMI <25, whereas an inverse association was observed for the group of BMI ≥25 women, with a *P* value for interactions  = 0.02. However, none of the risk estimates in each individual stratum in the analysis was significant. We did not find any statistically significant interactions for the remaining potential effect modifiers.

**Table 3:** Modification of associations between dietary cadmium intake and breast and endometrial cancer by relevant stratification factors.

		Breast cancer			Endometrial cancer		
Stratification factors	N cases	IRR^a^	95% CI	*P* b	IRR^a^	95% CI	*P* b
Education				0.77			0.68
Low (<8 y)	422	0.92	(0.72, 1.17)		1.02	(0.55, 1.87)	
Medium (8–10 y)	687	1.01	(0.84, 1.21)		1.14	(0.70, 1.86)	
High (>10 y)	281	1.03	(0.79, 1.35)		0.77	(0.37, 1.61)	
Smoking				0.24			0.95
Never	587	1.11	(0.92, 1.35)		0.98	(0.62, 1.53)	
Former	335	0.85	(0.66, 1.11)		1.00	(0.47, 2.14)	
Current	468	0.95	(0.77, 1.19)		1.11	(0.55, 2.24)	
HRT users				0.63			0.55
Never	522	1.00	(0.81, 1.22)		0.82	(0.47, 1.44)	
Former	174	1.14	(0.81, 1.60)		1.35	(0.67, 2.72)	
Present	694	0.95	(0.79, 1.14)		1.04	(0.62, 1.77)	
BMI				0.12			0.02
<25	719	1.08	(0.91, 1.28)		1.50	(0.94, 2.39)	
≥25	671	0.89	(0.73, 1.07)		0.69	(0.42, 1.12)	
Total zinc intake				0.85			0.58
< median	677	1.00	(0.81, 1.24)		0.85	(0.46, 1.55)	
≥ median	713	0.98	(0.82, 1.15)		1.04	(0.67, 1.62)	
Total iron intake				0.99			0.23
< median	667	1.00	(0.78, 1.26)		0.68	(0.35, 1.32)	
≥ median	723	0.99	(0.84, 1.17)		1.10	(0.71, 1.69)	

Abbreviations: IRR, incidence rate ratio; CI, confidence interval.

a Adjusted for educational level (<8 y; 8–10 y; >10 y), smoking status (never; former; current), number of births (0; 1–2; 3–8), age at first birth (years, continuous), HRT status (never; former; current), HRT use (years, continuous), age at menarche (years, continuous), BMI (continuous), height (cm, continuous), physical activity (MET score, continuous) and alcohol intake (yes/no and g/day, continuous).

BMI (continuous) was not including in the stratification analyses on BMI.

b *P* values for interaction.

Incidence rate ratios are per 10 µg dietary cadmium intake/day. Adjusted models results are shown.

Table 4 shows the risk estimates for breast cancer among *a priori* defined subgroups. We did not establish any significant associations between dietary cadmium intake and breast cancer for any of the subgroups.

**Table 4:** Incidence rate ratios of breast cancer (per 10 µg dietary cadmium intake/day) for relevant subgroups.

Subgroups	N cases	IRR^a^	95% CI
Never-smokers and BMI<25	282	1.23	(0.94, 1.60)
Never-smokers and never HRT users	237	0.95	(0.70, 1.30)
BMI<25 and never HRT users	224	1.11	(0.82, 1.47)
Never-smokers, BMI<25 and never HRT users	92	1.08	(0.69, 1.71)

Abbreviations: IRR, incidence rate ratio; CI, confidence interval.

a Adjusted for educational level (<8 y; 8–10 y; >10 y), smoking status (never; former; current), number of births (0; 1–2; 3–8), age at first birth (years, continuous), HRT status (never; former; current), HRT use (years, continuous), age at menarche (years, continuous), BMI (continuous), height (cm, continuous), physical activity (MET score, continuous) and alcohol intake (yes/no and g/day, continuous).

Adjusted model results are shown.

For breast cancer, risk analyses were also carried out for estrogen receptor classification (ER+ and ER-), progesterone receptor classification (PGR+ and PGR-) and for the two most frequent histology types (ductal and lobular) (Table 5). No significant associations were found, but a tendency towards a positive association with dietary cadmium intake was seen for lobular breast cancer.

**Table 5:** Incidence rate radios of breast cancer classifications (per 10 µg dietary cadmium intake/day).

Breast cancer classification	N cases^a^	IRR^b^	95% CI
Estrogen receptor			
Positive	981	1.00	(0.85, 1.15)
Negative	228	0.88	(0.62, 1.22)
Progesteron receptor			
Positive	405	0.85	(0.67, 1.09)
Negative	266	1.12	(0.84, 1.49)
Histology			
Ductal	1,026	0.98	(0.89, 1.13)
Lobular	172	1.12	(0.78, 1.59)

Abbreviations: IRR, incidence rate ratio; CI, confidence interval.

a In each analyses, we excluded those with no information on the classification under study (ER status, PGR status or histology). For the histology analyses we also excluded those being characterised with other histology types than ductal and lobular.

b Adjusted for educational level (<8 y; 8–10 y; >10 y), smoking status (never; former; current), number of births (0; 1–2; 3–8), age at first birth (years, continuous), HRT status (never; former; current), HRT use (years, continuous), age at menarche (years, continuous), BMI (continuous), height (cm, continuous), physical activity (MET score, continuous) and alcohol intake (yes/no and g/day, continuous).

Adjusted model results are shown.

## Discussion

In this study, we did not find significant associations between dietary cadmium intake and risk of hormone-related cancers in postmenopausal women. In line with our results, the large American prospective VITAL cohort study did not find any evidence of an association between dietary cadmium intake and postmenopausal breast cancer risk [20]. Similarly, a prospective Japanese study did not find an association between dietary cadmium intake and breast and endometrial cancer [21] and a Japanese case-control study did not find a significant association between dietary cadmium intake and risk of breast cancer [29]. In contrast to these and to our results, prospective studies of postmenopausal women in the Swedish Mammography Cohort (SMC) reported positive associations with postmenopausal endometrial cancer [22] and breast cancer [19], but did not find an association for ovarian cancer [23]. In spite of the fact that these studies (19–22, 29) and our study are relatively similar in exposure assessment and in study design, they represent four somewhat different countries with differences in dietary habits, quality of cadmium monitoring data, pollution levels of cadmium, etc., which could have impacted these studies differently. Still there is consistency in findings (except for SMC's positive association for endometrial and breast cancer). The above described studies included a wide range of dietary cadmium exposure with mean values ranging from 10.9 µg/day in the American study [20] to 26.4 µg/day in the Japanese study [29], whereas in Sweden similar values were found as in our study [22]. That is, based on the present literature, dietary cadmium intake does not overall seem to be associated with risk of hormone-related cancers in postmenopausal women, regardless of exposure levels. Whether this picture reflects a true lack of association with hormone-related cancers or whether the results reflect non-differential exposure measurement error in the estimation of dietary cadmium intake concealing a true association is unclear.

Two American retrospective breast cancer case-control studies found significant trends for increased odds ratios across quartiles of urinary cadmium levels [30]–[31]. Urinary cadmium level is considered the standard biomarker for lifetime cadmium body burden in the general population and this procedural contrast to our study may to some extent clarify the discrepancy in results. However, a limitation of the case-control studies is that urine samples were collected after diagnosis, introducing the possibility of disease- or treatment-related alterations of the urine cadmium measure, leading to a non-causal association between urinary cadmium measurement and cancer risk.

The clinical, pathologic, and molecular characteristics of breast cancer differ by their ER and/or PGR expression profile and the effects of risk factors of breast cancer, such as reproduction related exposures, also differ by ER/PGR status [32]. Cadmium has been shown to bind the nuclear ER and appears to interact with its hormone-binding domain [12]. Recently, cadmium was shown to activate membrane-bound ERs [33], indicating an alternative mode of action even in the absence of nuclear ER. Examining the association between cadmium exposure and ER as well as PGR subtypes may provide further insights into possible hormone disrupting properties of cadmium. In this study, we investigated whether the association between cadmium and breast cancer risk differed between ER and PGR expression and histology. A previous study found significant positive association among ER+ and PGR- patients [29], but we could not confirm these findings in our study. Our results showed a relatively stronger association for the lobular breast cancer subtype compared with the ductal subtype, though none of the results were significant. Dietary cadmium intake has to the best of our knowledge not been investigated as a risk factor for histological subtypes before and for PGR status previously only in one case-control study [29].

After menopause, women with higher BMI have a slightly increased risk of breast cancer compared with leaner women. This may be explained by the fact that estrogen after menopause is formed mainly in the adipose tissue. Obese postmenopausal women have plasma levels of endogenous estrogens nearly twice as high as lean women [2].We conducted analyses stratified by BMI and expected *a priori* to obtain a higher estimate for women with lower BMI due to the reduced influence of adipose tissue-derived estrogen exposure. We found an interaction for endometrial cancer and to a lesser extent for breast cancer. This tendency was also seen in other [19], [22], but not all [20], [29] studies. Since tobacco smoking is a source of cadmium intake, it has often been assumed that the lack of positive findings for tobacco on breast cancer indicates that cadmium is not a risk factor [34], although smoking also has some anti-estrogenic properties [35] perhaps masking a cadmium effect. In support of the anti-estrogenic effect, smoking has been associated with decreased risk of endometrial cancer. In order to minimize the potential effect of exposure to endogenously produced adipose tissue estrogen, obtained hormones from medical treatment and/or to smoking-derived cadmium, we performed analyses stratified by BMI, HRT and smoking. We investigated all combinations of being never smokers, having BMI<25 and being never HRT users, but did not find statistically significant associations.

Cadmium shares some structural similarities with the mineral zinc and there is some mechanistic evidence that zinc increases the sequestering of cadmium by inducing metal-binding metallothioneins, as well as directly reducing cadmium absorption [36]–[37]. The iron-cadmium ratio also seems to be important since low body iron stores seem to be linked to increased intestinal absorption of cadmium [38]. Therefore, we would expect the strength of a possible association between dietary cadmium intake and cancer to be most prominent among women with low levels of zinc intake or iron intake. However, neither zinc nor iron seemed to modify the association between cadmium intake and cancer risk. These null results were also found in the American study on dietary cadmium and breast cancer risk, which did not find evidence for interactions between cadmium and breast cancer risk factors, smoking habits, iron or zinc intake [20]. However, mechanistic evidence of interplay suggests the role of cadmium versus zinc and iron to be investigated further in epidemiologic studies of hormone-related cancers.

The major strength of this study is the prospective design that was based on a well-defined cohort with data on potential confounders. Furthermore, virtually complete nationwide registries provided information on vital status and cancer status. Further, disease status could not have biased exposure assessment because questionnaire data was obtained before cancer diagnosis.

Non-differential exposure measurement error in the estimation of dietary cadmium intake may be a factor concealing a true association between cadmium and the investigated cancers. That is, a limitation of this study includes our ability to accurately assess dietary cadmium intake, which may have moderated our estimates. Participants of the Diet, Cancer and Health cohort were asked to report their average dietary habits within the year prior to enrolment, and these answers may reflect long-term dietary pattern and long-term exposure to dietary cadmium. However, their dietary pattern may have changed during the follow up period. Also, some deviation in the content of cadmium in specific food items could be another important source of measurement error. Future studies are needed including use of urinary levels as marker for cadmium exposure in relation to hormone-related cancer in large prospective studies.

In conclusion, the results of the present study do not support the hypothesis that cadmium contamination of food is a risk factor for postmenopausal hormone-related cancers in women.
